# Palaeoproteomic identification of breast milk protein residues from the archaeological skeletal remains of a neonatal dog

**DOI:** 10.1038/s41598-019-49183-0

**Published:** 2019-09-06

**Authors:** Takumi Tsutaya, Meaghan Mackie, Claire Koenig, Takao Sato, Andrzej W. Weber, Hirofumi Kato, Jesper V. Olsen, Enrico Cappellini

**Affiliations:** 1 Research Institute for Marine Resources Utilization, Japan Agency for Marine-Earth Science and Technology, Natsushima 2-15, Yokosuka, Kanagawa 237-0061 Japan; 2 Section for Evolutionary Genomics, The GLOBE Institute, University of Copenhagen, Øster Voldgade 5–7, 1350 Copenhagen, Denmark; 3 Proteomics Program, Novo Nordisk Foundation Center for Protein Research, Faculty of Health Science, University of Copenhagen, Blegdamsvej 3b, 2200 Copenhagen, Denmark; 4 European School of Chemistry, Polymers and Materials Science, University of Strasbourg, 25 rue Becquerel, 67087 Strasbourg, France; 5 Department of Archaeology and Ethnology, Faculty of Letters, Keio University, Mita 2-15-45, Minato, Tokyo 108-8345 Japan; 6 Department of Anthropology, 13-15H.M. Tory Building, University of Alberta, Edmonton, Alberta T6G 2H4 Canada; 7 Laboratoire Méditerranéen de Préhistoire Europe Afrique (LAMPEA)—UMR 7269, Aix-Marseille Université, 5 rue du Château de l’Horloge—BP 647, 13094, Aix-en-Provence, Cedex 2 France; 8 Department of History, Irkutsk State University, Karl Marx Street 1, Irkutsk, 664003 Russia; 9 Center for Ainu and Indigenous Studies, Hokkaido University, Sapporo, Kita 8, Nishi 6, Kita-ku, 060-0808 Japan

**Keywords:** Proteomics, Archaeology, Proteomics

## Abstract

Accurate postmortem estimation of breastfeeding status for archaeological or forensic neonatal remains is difficult. Confident identification of milk-specific proteins associated with these remains would provide direct evidence of breast milk consumption. We used liquid chromatography coupled to tandem mass spectrometry (MS) to confidently identify beta-lactoglobulin-1 (LGB1) and whey acidic protein (WAP), major whey proteins associated with a neonatal dog (*Canis lupus familiaris*) skeleton (430–960 cal AD), from an archaeological site in Hokkaido, Japan. The age at death of the individual was estimated to be approximately two weeks after birth. Protein residues extracted from rib and vertebra fragments were analyzed and identified by matching tandem MS spectra against the dog reference proteome. A total of 200 dog protein groups were detected and at least one peptide from canine LGB1 and two peptides from canine WAP were confidently identified. These milk proteins most probably originated from the mother’s breast milk, ingested by the neonate just before it died. We suggest the milk diffused outside the digestive apparatus during decomposition, and, by being absorbed into the bones, it partially preserved. The result of this study suggests that proteomic analysis can be used for postmortem reconstruction of the breastfeeding status at the time of death of neonatal mammalian, by analyzing their skeletal archaeological remains. This method is also applicable to forensic and wildlife studies.

## Introduction

Breastfeeding is one of the most important factors for the survival of mammalian infants, because breast milk provides immunological benefits and precious nutrients^1^. Infants that grow up without breastfeeding are statistically prone to higher mortality^2^. Therefore, accurate estimation of breastfeeding status is important to investigate the cause of infant mortality in mammals. Although behavioral observation and doubly labeled water methods are used to estimate breastfeeding status of living individuals^3^, these methods cannot be applied to postmortem estimation.

Stable isotope analysis has been used for the estimation of breastfeeding status in modern and ancient biological tissues from several mammalian species^4^. This method, however, cannot be reliably applied to identify the breastfeeding status of neonates, because several weeks or months are required for given tissues to reflect the isotopic signal of the diet. Accordingly, the isotopic signal of breastfeeding is hardly detectable in neonates who die soon after birth. Furthermore, nitrogen isotope ratios are affected by other factors than breastfeeding and weaning^5^, and the increased nitrogen isotope ratios in infants are not necessarily direct evidence of breast milk consumption.

To overcome these limits, we attempted reconstruction of the breastfeeding status in neonates, by directly detecting milk-specific proteins still associated with archaeological neonatal skeletal remains. We hypothesized that, in a neonate who died after ingesting milk through breastfeeding, milk proteins would diffuse from the digestive system into the surrounding bones during decomposition. Consequently, we tested whether these milk proteins can be confidently identified by tandem mass spectrometry (MS)-based sequencing, assuming the infant remains did not experience remarkable postmortem disturbances.

Recently, palaeoproteomic analysis has enabled comprehensive identification of even low amounts of proteins and peptides in ancient biological tissues^6^. Proteomic analysis by liquid chromatography coupled to tandem mass spectrometry (LC-MS/MS) has been used in bioarchaeological and evolutionary studies, such as reconstruction of protein expression profiles^7^, phylogeny^8,9^, diet^10,11^, and physiological status^12,13^, as well as species^14,15^ and sex^16^ identification. Palaeoproteomics also allowed detection of dinosaur proteins^17^, even though some caution has been raised toward these results^18^. While dietary reconstruction by proteomic analysis has targeted food remains^11,19^ or dental calculus^10,20^, we report the detection of breast milk-specific proteins from ancient neonatal dog bone remains.

## Materials and Methods

### Hamanaka 2 site and the dog neonate

A neonatal dog (*Canis lupus familiaris*) skeleton (2017HA1016) was excavated from the Nakatani location of the Hamanaka 2 site, Rebun Island, Hokkaido, Japan (Supplementary Fig. 1). Hamanaka 2 site is a multi-component shell midden, spanning from final Jomon (3000−2300 years BP) to historical Ainu (400 years BP) periods. The cool temperature of Rebun Island (mean annual temperature in the period 1978–2002 was 6.6 °C^21^) and the presence of shell promotes good preservation of organic materials at the Hamanaka 2 site. Since 2011, archaeological excavations have yielded human remains, rich faunal remains, lithics and pottery fragments^22–25^.

The 2017HA1016 remains, originating from the Kokumon subperiod of the Okhotsk cultural layer (430–960 cal AD^22^) and partially retaining their anatomical articulation, were found in 2017. Excavation and collection of the 2017HA1016 skeletal remains, as well as of an adjacent fish bone from the same context, was done wearing a face mask and nitrile gloves. Additional bones were recovered using a 4 mm mesh. Some of these specimens were frozen within one hour after excavation (see Table 1). The age at death of 2017HA1016 was estimated using the reference chart of tooth development and eruption for modern Japanese dogs^26^.

**Table 1:** Detail of the analyzed samples.

Element	Position	Sample handling history	Analytical ID	Fraction
Fish bone	—	Frozen until analysis	1016F-E	EDTA
			1016F-P	Pellet
Dog vertebral bone	Vertebral body	Frozen until analysis	1016V-E	EDTA
			1016V-P	Pellet
Dog rib bone 1	Distal half	Frozen until analysis	1016R1d-E	EDTA
			1016R1d-P	Pellet
	Proximal half	Kept in room temperature	1016R1p-E	EDTA
			1016R1p-P	Pellet
Dog rib bone 2	Mid-shaft	Kept in room temperature	1016R2	EDTA + Pellet
Dog rib bone 3	Proximal end	Kept in room temperature	1016R3	EDTA + Pellet
Soil	—	Kept in room temperature	1016 S	—

Blanks were also present.

### Palaeoproteomic analysis

Ancient proteins were extracted from an entire 2017HA1016 vertebra body and three rib fragments. As negative controls, a fish bone and a soil specimen, collected less than 10 cm away from the 2017HA1016 remains, were also processed using the same experimental workflow (Table 1). Details of the proteomic methodology are reported as Supplementary Information. Briefly: bones were decalcified with EDTA solution and the proteins in the EDTA supernatant and in the collagenous pellet were separately denatured^27^, reduced, and alkylated (adapted from^28^). Protein solutions were then digested using trypsin overnight at 37 °C. Tryptic peptides from both fractions of each bone extract were purified using Stage Tips with C18 membrane^29^ and analyzed separately, unless otherwise specified, by nanoflow liquid chromatography tandem mass spectrometry (nLC-MS/MS), using an EASY-nLC 1200 connected to a Q-Exactive HF-X (ThermoFisher, Bremen - Germany).

RAW data files generated by LC-MS/MS were searched against a *Canis lupus familiaris* proteome database downloaded from Uniprot, and a common laboratory contaminant database, with the MaxQuant software version 1.5.3.30^30^. Protein groups having at least 2 different non-overlapping peptides were considered confidently identified, unless otherwise indicated. All protein hits that could be considered possible contamination products were excluded from further analysis. Deamidation rates for individual samples were calculated with a Python script^15^. Detected proteins were classified using PANTHER database version 13.1^31^.

## Results

### Morphology of the dog neonate and estimation of its age at death

**Figure 1:**
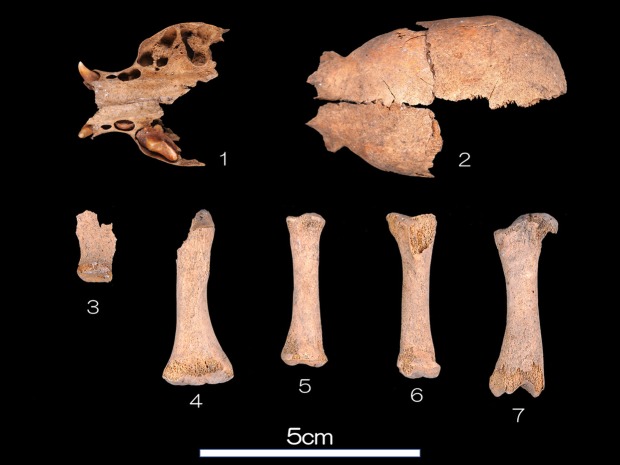
Photo of the main bones of neonatal dog skeleton (2017HA1016): (1) maxilla and frontal; (2) parietal; (3) scapula R; (4) humerus L; (5) radius R; (6) tibia R; (7) femur R.

As the deciduous first molar does not erupt while the deciduous canine and deciduous second molar are erupting in the maxilla, the age at death of 2017HA1016 was estimated at 2 weeks after birth, based on the Mori’s reference chart^26^ (Fig. 1). Most elements of the skeleton were preserved, and the recovered bones of the skeleton are described in Supplementary Table 1.

### Detected proteins

Only collagen α-1(I) fragments (COL1A1) were detected in the fish bone by searching against the dog proteome, and only actin cytoplasmic 1 (ACTB) and COL1A1 were detected with ≥2 non-overlapping peptides in the soil sample. The following results and discussion are therefore limited to the data obtained from the analysis of the bone fragments of the 2017HA1016 dog. Protein groups identified in blanks but not in the dog bones with ≥2 peptides were not considered.

A total of 200 protein groups were detected across the EDTA and pellet fractions of the rib and vertebra bones (Supplementary Table 2). A total of 143 protein groups (71.5%) were detected in both the EDTA and the pellet fractions, and 154 protein groups (77.0%) were retrieved from both rib and vertebra. Recovered proteomes can differ in multiple experimental fractions extracted from a single sample^32^ and from different bone elements from a single individual^33^. PANTHER analysis indicated that 51.6% of protein groups are classified as an extracellular component (Supplementary Table 3) and 41.3% are related with binding function (Supplementary Table 4). Mean deamidation rates of samples were 36.3 ± 4.8% for asparagine (N) and 11.0 ± 2.1% for glutamine (Q) (n = 8; Supplementary Table 5). Deamidation of glutamine and asparagine residues is a non-enzymatic modification that occurs over time, resulting in a + 0.98402 Da mass shift caused by the direct or indirect hydrolysis of the N and Q side-chain amide group^34^.

### Biological marker proteins

Protein groups characteristic of fetuses or newborns were detected from bones of 2017HA1016 (Table 2). Alpha-fetoprotein (AFP), a major plasma protein expressed in yolk sac and fetal liver and only detected in fetuses or young neonates^35^, was found in all of the four bones. AFP may play a role comparable to that of serum albumin in the adult, and its concentration is at its highest just after delivery (14080 ± 5944 μg/mL) and rapidly decreases during the first 2 weeks after birth (70.21 ± 52.92 μg/mL) in dogs^35^.

**Table 2:** Characteristic protein groups for fetus or growing infant and milk that were detected in 2017HA1016.

Gene name	Protein name	Protein ID	Number of unique peptides	Sequence coverage	Score	≥2 peptides detected in
AFP	Alpha-fetoprotein	F1PXN2	28	38.4	213.49	R1d-E, R1d-P, R1p-E, R1p-P, R2, R3
COL10A1	Collagen type X alpha 1 chain	J9P1I7	8	13.2	42.03	V1-E, V1-P, R1d-E, R1p-E, R1p-P
EPYC	Epiphycan	E2R430	3	11.2	46.33	R1p-E
LGB1	Beta-lactoglobulin-1	P33685	2	12.4	4.22	R1p-E
WAP	Whey acidic protein	H9GW77	2	15.4	4.97	R1d-E

Several protein groups that are related with endochondral bone formation were detected (Table 2). Most bones, including ribs and vertebrae, develop via endochondral bone formation; endochondral cartilage templates are then replaced by calcified bone matrix^36^. Collagen type X, alpha-1 (COL10A1) has functions in bone formation, and is only expressed in endochondral cartilage that will be replaced by mature bone tissue in humans^37^. Epiphycan (EPYC) is a small leucine-rich proteoglycan that is mostly found in fetal and neonatal epiphyseal cartilage in mice, bovines, and chickens^38^. EPYC has important roles in cartilage development and its maintenance^39^. Detection of these proteins (Table 2) is consistent with the age at death of the 2017HA1016 individual and it confirms that palaeoproteomics can retrieve growth- or developmental stage-specific proteins from archaeological bone remain^28,40^.

**Figure 2:**
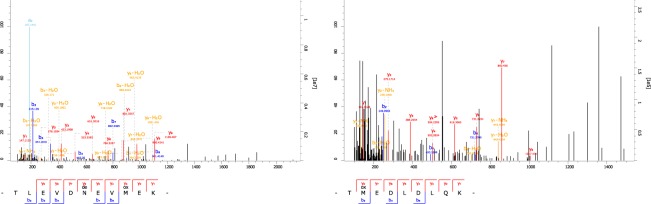
MS2 spectra of peptides assigned to LGB1.

In addition, two milk proteins were detected from the EDTA fractions of two distinct subsamples, i.e. the distal and proximal ends of a single rib. Two peptides of beta-lactoglobulin-1 (LGB1) were detected from the EDTA fraction of the distal part of 2017HA1016’s rib (Fig. 2, Table 3). LGB1 is a major whey protein, absent in some mammalian species, including humans^41^. Although its exact physiological function is not determined yet, LGB1 binds several hydrophobic ligands and thus may act as specific transporters^41^.

**Table 3:** Matched peptides for milk proteins.

Protein	Peptide	Posterior error probability	Score
Beta-lactoglobulin-1	TLEVDNEVMEK	0.000176	101.43
	TMEDLDLQK	0.025502	60.30
Whey acidic protein	SCVVPFIVPVQK	0.000470	97.45
	CCLSVCAMR	0.001726	100.09

Protein BLAST searches indicated that the two detected peptides of LGB1 sequences were identical to those of dogs with the highest score. One of the detected peptide sequences (TLEVDNEVMEK) also matches the sequence of glycodelin of *Vulpes vulpes* (red fox). The identification of the other sequence (TMEDLDLQK) is supported by a spectrum including the complete y-ion series (Fig. 2), however, it’s  quality is lower, most probably due to random co-fragmentation of the precursor with another peptide. This spectrum could also be assigned equally confidently to the deamidated (N → D) peptide sequence of LGB from *Callorhinus ursinus* (northern fur seal), *Odobenus rosmarus divergens* (Pacific walrus), and *Leptonychotes weddellii* (Weddell seal). However, its assignment to dog LGB1 represents the most parsimonious interpretation.

**Figure 3:**
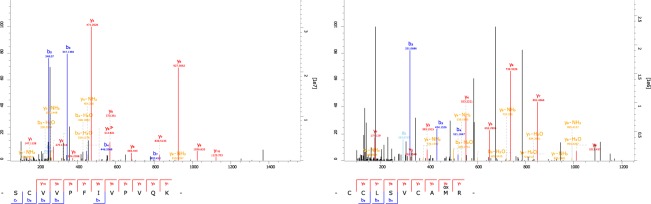
MS2 spectra of peptides assigned to WAP.

In addition, two peptides of whey acidic protein (WAP) were detected from EDTA fraction of the proximal part of 2017HA1016’s rib (Fig. 3, Table 3). WAP is a major whey protein present in dog milk, which seems to play important roles in regulating the proliferation of mammary epithelial cells^42^. The detected peptides of WAP perfectly match with the dog WAP sequence reported by Seki and colleagues^42^. Protein BLAST searches indicated that the sequences recovered uniquely match dog WAP. Unassigned higher peaks in MS2 spectrum of CCLSVCAMR (Fig. 3) were mostly originated from neutral losses (Supplementary Fig. 2).

Since the neonatal dog is too young to secrete breast milk, the detected peptides of LGB1 and WAP would most probably originate from its mother. The alternative origin of LGB1 and WAP from the above-mentioned non-dog species is archaeologically and ecologically highly unlikely. The possibility of contamination is excluded because no LGB1 and WAP peptides were detected in the negative controls. Finally, the only species that matches all observed peptides is *Canis lupus familiaris*.

We also detected protein groups that are expressed in milk, but also in other tissues. Milk fat globule-EGF factor 8 (MFGE8) facilitates scavenging of the dying cells from the tissue and is an essential factor for controling the progression of various inflammatory diseases^43^. MFGE8 is a component of milk fat globule membrane protein, but it is also expressed ubiquitously in other cells and tissues^43^. Fourteen peptide-spectrum matches of MFGE8 were obtained from the dog bone samples.

## Discussion

### Authenticity of the recovered proteome

The detection of specific proteins exclusively expressed in neonates^14^ in 2017HA1016 (Table 2) is consistent with the morphological observation that confidently established this individual died approximately two weeks after birth. In particular, AFP^35^ and EPYC^38,39^ are expressed in fetal liver and endochondral cartilage, respectively. COL10A1 is present only in ossifying endochondral cartilage and will be replaced in mature bone tissue^37^. This evidence supports the authentic endogenous origin of the palaeoproteome retrieved.

We also measured relatively high rates of asparagine (36.3 ± 4.8%) and glutamine (11.0 ± 2.1%) deamidation (n = 8; Supplementary Table 5), compared to modern similar samples^34^. This observation is compatible with the endogenous ancient origin of the peptides we detected in the dog neonate, as high deamidation rates are generally correlated, in archaeological samples, with prolonged postmortem protein degradation^34,44^ (but see^45^).

### Detection of breast milk proteins

Although the original articulation of the 2017HA1016 skeleton was retained only partially, there was no evidence indicating the burial was affected by diagenetic or ritual manipulation. No other dog remains were found around 2017HA1016. Similarly, no archaeological or ethnographic evidence of human exploitation and consumption of domesticated dog milk is documented in Hokkaido. Additionally, the negative controls processed in parallel to the dog bone samples did not show any evidence of canine milk proteins. Therefore, it is reasonable to conclude that the detected LGB1 and WAP peptides do not represent contamination products derived from anthropogenic or postmortem processes.

After excluding the possibility that the dog-specific LGB1 and WAP peptides originate from contamination, as well as anthropogenic or diagenetic activities, we conclude they originated from the breast milk the neonate ingested just before dying. During the following decomposition, the milk eventually diffused from the digestive system and was absorbed into the bone tissue. Neonatal dogs consume a large amount of breast milk, with consumption reaching its peak at 3–4 weeks after birth^46^. At 19 and 26 days after birth, breast milk represents respectively 17.0% and 14.6% of the animal body mass^46^. It is highly plausible that 2017HA1016 consumed such large amounts of breast milk just before its death, and consequently that some protein residues from such a relatively large amount of undigested milk diffused inside the body of the decomposing neonate dog soon after its death. Those protein residues eventually reached some districts of the dog skeleton where, by complexing with the mineral bone matrix, they were preserved until detected by MS analysis. It has been repeatedly observed that ancient protein preservation is higher for those residues tightly bound to the mineral matrix of bones, dental enamel, or eggshells^10,20,47,48^. This mechanism seems to represent a key factor in reducing spontaneous ancient protein backbone hydrolysis over extremely long time intervals^47,48^. Being the milk protein most frequently retrieved in archaeological human dental calculus^20^ and pottery matrix^47^, LGB1 is consistently preserved relatively better than other proteins in archaeological samples. Interestingly, the ruminant homologs of one of the LGB1 peptides (TLEVDNEVMEK) detected in this study, such as TPEVDDEALEK in bovine LGB, represent the most frequently detected LGB peptides (n = 70/135) in Bronze Age human dental calculus^49^. Both the TLEVDNEVMEK and the TPEVDDEALEK peptides are particularly rich in acidic amino acids, i.e. D, E and deamidated N, which are known to directly bind biominerals, enabling ancient peptide recovery even after millions of years in climatically adverse environments^48^. Most probably such a mechanism favoured the preservation of the ancient breast milk peptides we detected in association with the 2017HA1016 skeletal remains.

The MFGE8 protein was also detected in several samples. Studies with human infants show that there is a much higher concentration of this protein in the gut of those neonates fed with maternal milk compared to bottle-fed ones^50^, indicating that there is a definite transfer of this protein from the mother through her breast milk. MFGE8, however, has also been detected in archaeological adult human^28^ and mammoth^7^ bones. Detection of proteins not exclusively expressed in breast milk is therefore indicative, but not conclusive, evidence of milk consumption.

### Proteomic reconstruction of breastfeeding status

To the best of our knowledge, this is the first study that reports the survival of ingested breast milk proteins in ancient skeletal material. The results of this study demonstrate that ingested maternal breast milk can be absorbed by the bones during postmortem decomposition and detected using proteomic analysis.

This study shows that the breastfeeding status of a neonate can be reconstructed by applying proteomic analysis to its archaeological remains. Proteomic estimation of breastfeeding status has three advantages compared to isotopic and observational analyses: (i) it can be applied to dead individuals whose behavior cannot be observed, (ii) it offers direct evidence of breast milk consumption, and (iii) it can provide species-specific identification of the milk source. This method is not only applicable to archaeological samples but has the potential to be used in forensic and wildlife studies.

Proteomics-based estimation of breastfeeding status is associated with some limitations as well. First, milk proteins might not be detected from the remains of biologically older individuals who, despite dying while they were breastfed, consumed proportionally smaller amounts of milk in relation to their body mass. For example, in a previous proteomic study, no milk protein was detected in a rib bone from an archaeological human skeleton of a nine months old individual^28^, despite the cessation of breastfeeding was reconstructed to occur most probably at the age of 3.1 years in that population^51^. Second, this method is harder to apply to remains from mammalian species whose genome and/or reference proteome is not publicly available yet.

## Conclusions

Dog milk (LGB1 and WAP) and fetal/infant marker (AFP, COL10A1, and EPYC) proteins were detected in rib and vertebra bones of an ancient (430–960 cal AD) dog neonate (2017HA1016) that died 2 weeks after birth. The dog milk proteins most probably originated from the mother’s breast milk. This is the first study that reports the survival of ingested breast milk proteins in an ancient mammalian skeleton.

## Supplementary information

Supplementary Information

Supplementary Table 2

Supplementary Table 6

Supplementary Table 7

## Data Availability

RAW data have been uploaded to ProteomeXchange^52^ with the dataset identifier PXD014657.
