# Supplementary material for: Palaeoproteomic identification of breast milk protein residues from the archaeological skeletal remains of a neonatal dog
Source: Sci Rep. 2019 Sep 6;9:12841. doi: 10.1038/s41598-019-49183-0 (PMC6731306; doi:10.1038/s41598-019-49183-0)
Supplement: Supplementary file 1 — Supplementary Information [file 41598_2019_49183_MOESM1_ESM.pdf]

Supplementary Information

Palaeoproteomic identification of breast milk protein residues from the archaeological skeletal remains of a neonatal dog

Takumi Tsutaya[1*], Meaghan Mackie[2,3], Claire Koenig[4], Takao Sato[5], Andrzej W Weber[6,7,8], Hirofumi Kato[9], Jesper V Olsen[3], Enrico Cappellini[2]

[1] Research Institute for Marine Resources Utilization, Japan Agency for Marine-Earth Science and Technology, Natsushima 2-15, Yokosuka, Kanagawa, 237-0061, Japan.

[2] Section for Evolutionary Genomics, The GLOBE Institute, University of Copenhagen, Øster Voldgade 5–7, 1350 Copenhagen, Denmark.

[3] Proteomics Program, Novo Nordisk Foundation Center for Protein Research, Faculty of Health Science, University of Copenhagen, Blegdamsvej 3b, 2200 Copenhagen, Denmark.

[4] European School of Chemistry, Polymers and Materials Science, University of Strasbourg, 25 rue Becquerel, 67087 Strasbourg, France

[5] Department of Archaeology and Ethnology, Faculty of Letters, Keio University, Mita 2-15-45, Minato, Tokyo, 108-8345, Japan.

[6] Department of Anthropology, 13-15 H.M. Tory Building, University of Alberta, Edmonton, Alberta T6G 2H4, Canada.

[7] Laboratoire Méditerranéen de Préhistoire Europe Afrique (LAMPEA)—UMR 7269, Aix-Marseille Université, 5 rue du Château de l'Horloge—BP 647, 13094 Aix-en-Provence Cedex 2, France.

[8] Department of History, Irkutsk State University, Karl Marx Street 1, Irkutsk 664003, Russia.

[9] Center for Ainu and Indigenous Studies, Hokkaido University, Sapporo, Kita 8, Nishi 6, Kita-ku, 060-0808, Japan

[*] Corresponding author: Tsutaya T. Tel.: +81-46-867-9857; E-mail: tsuta@jamstec.go.jp or tsutayatakumi@gmail.com

ORCID: TT, 0000-0001-5104-5223; MM; 0000-0003-0763-7592; EC, 0000-0001-7885-7811

Proteomic methodology

(a) Protein extraction and proteomic analysis

Proteins were individually extracted from approximately 20−30 mg of a full vertebra body and ribs of 2017HA1016 and approximately 20−30 mg of the associated fish bone and soil sample (Cleland, 2018; Sawafuji et al., 2017). Bones were decalcified by 0.5 M EDTA solution over 24 h. Proteins in the EDTA supernatant were collected using Amicon Ultra Centrifugal Filters (molecular weight cutoff of 3000 Da) and denatured, reduced, and alkylated in 2M guanidinium hydrocloride (GuHCl) solution containing 10 mM tris(2-carboxyethyl)phosphine hydrochloride (TCEP) and 20 mM 2-chloroacetamide (CAA), in concurrence with an additional EDTA blank control. The pellet leftover from decalcification was suspended in the same 2M GuHCl solution, heated at 80°C for 2 h to denature collagen fibrils, and proteins were denatured, reduced, and alkylated, along with a pellet blank control. Soil samples were suspended in 6M GuHCl solution, containing 10 mM TCEP and 20 mM CAA, in concurrence with an additional blank control. After heating at 99°C for 10 min, the solutions were centrifuged (2500g, 5min) to pellet insoluble material. The supernatants were collected and centrifuged again (17000g, 60-120 min). The collected supernatants were fractionated to obtain proteins using an Empore reverse-phase C-8 cartridge with 40% and 60% acetonitrile (ACN) + 0.5% acetic acid. The fractionated samples were vacuum-dried, and suspended into the 2M GuHCl solution containing 10 mM TCEP and 20 mM CAA. The concentrations of resultant protein solutions of each fraction were measured by Bradford assay. Protein solutions were then digested with 0.2 µg LysC (Promega, Sweden) for 1.5 h at 37°C and then diluted with 25 mM Tris in 10% ACN to the final concentration of 0.6 M GuHCl. Protein solutions were digested again using 0.8 µg trypsin (Promega, Sweden) overnight at 37°C. Digested solutions were acidified by 10% trifluoroacetic acid (TFA) and centrifuged to pellet any non-protein components. Peptides in the supernatant were purified and desalted using in-house made StageTips with C18 membrane (Rappsilber et al., 2007).

Samples were eluted from the StageTips using 20 µl 40% ACN in water and then 10 µl 60% ACN into a 96 well MS plate. Samples were placed in a vacuum centrifuge at 40°C until approximately 3 µl of solution was left and then rehydrated with 5 µl of 0.1% TFA and 5% ACN solution.

Samples were then separated on a 15 cm column (75 µm inner diameter) which is made in-house, laser pulled, and packed with 1.9 µm C18 beads (Dr. Maisch, Germany) on an EASY-nLC 1000 (Proxeon, Odense, Denmark). The column was connected to a Q-Exactive HF-X (Thermo Scientific, Germany) on a 77 min gradient. Buffer A was milliQ water, and the peptides were separated with increasing buffer B (80% ACN and 0.1% formic acid), going from 5% to 30% in 50 min, 30% to 45% in 10 min, 45% to 80% in 2 min, held at 80% for 5

min before dropping back down to 5% in 5 min and held for 5 min. Flow rate was 250 nl/min. The column temperature was kept at 40°C. A wash-blank method using 0.1% TFA and 5% ACN solution was run in between each sample to hinder cross-contamination.

The Q-Exactive HF-X was operated in data dependent top 10 mode. Spray voltage was 2 kV, S-lens RF level was 50, and capillary was heated at 275°C. Full scan mass spectra were recorded at a resolution of 120,000 at m/z 200 over the m/z range 350–1400 with a target value of 3e6 and a maximum injection time of 25 ms. HCD-generated product ions were recorded with a maximum ion injection time set to 118 ms and a target value set to 2e5 and recorded at a resolution of 60,000. Normalized collision energy was set at 28% and the isolation window was 1.2 m/z with the dynamic exclusion set to 20 s.

(b) Data analysis

RAW data files generated by LC-MS/MS was searched against a *Canis lupus familiaris* proteome database downloaded from UniProt (as of 2019-06-22) with MaxQuant software version 1.5.3.30 (Cox and Mann, 2008). The following parameters were used for the analysis: parent mass error and fragment mass tolerances were those preset for Orbitraps. Carbamidomethylation was set as a fixed modification, and oxidation of methionine, deamidation of Asparagine and Glutamine, derivation of pyroglutamic acid, and hydroxylation of Proline were set at variable modifications. Up to a maximum of 5 modifications per peptide was allowed. All peptides were automatically filtered by a false discovery rate (FDR) of 1.0% and manually filtered by at least 2 different non-overlapping peptides. All contaminant accessions (i.e., keratins and trypsin) were excluded from further analysis using the contamination.fasta provided by MaxQuant, which includes common laboratory contaminants.

Deamidation rates for Asparagine and Glutamine for individual samples were calculated by using a program that works on Python software (Mackie et al., 2018, available at https://github.com/dblyon/deamidation). Because the deamidation rate is correlated, to a certain extent, with postmortem age of the protein, higher rate of deamidation is one of the evidences of authenticity of ancient proteins (Van Doorn et al. 2012). Briefly, the deamidation rates for each peptide-to-spectrum match (PSM) was calculated with a weight of “Intensity” of the given PSM. Deamidation rate per sample was calculated from the average of the peptide level deamidation rate after excluding contaminant peptides. The mean, the standard deviation, and the 95% confidence intervals of the deamidation rate per sample were calculated to obtain an estimate of the error of the calculation with 1000-times-bootstrapping.

The proteome of modern adult dog bone, derived from the cortical part of a diaphyseal humerus that was reported in Cleland (2018) was used as a comparative dataset. The RAW

files reported in Cleland (2018) were reanalyzed using MaxQuant with the same parameters described above, except for no fixed modification for solid digested samples without reduction and alkylation. Protein groups that have at least 1 unique peptide was considered as present, following the original definition by Cleland (2018).

Detected proteins were classified using PANTHER database version 13.1 (Mi et al., 2013).

Alignment of dog LGB1 (UniProt ID: P33685) with the bovine LGB (*Bos taurus*, UniProt ID: P02754) sequences was done by using EMBOSS Needle program.

In order to exclude the possibility that the peptide-spectrum matches of the milk proteins were the result of contamination by soil bacteria, RAW data files of the fractions where milk proteins were identified (1016R1d-E and 1016R1p-E) and soil sample (1016S) were also searched against a Swiss-Prot bacterial protein database (downloaded at 2019-06-20) and the dog proteome database. The analytical parameters were the same with those described above.

Supplementary Results and Discussion

(a) Proteins that were found from experimental blanks

Several protein groups (e.g., collagen, alpha 2-HS glycoprotein, and serum albumin) that are not listed as laboratory contaminant are also detected in experimental blanks (Supplementary Table 2). Although it is possible that these protein groups are contaminants, their numbers of detected peptides from experimental blanks were substantially lower than those from samples (Supplementary Table 2). Therefore, these protein groups were still included in the analysis, but should be treated with caution.

Although some protein groups were also detected in the experimental blanks, the protein groups that were described in the Result and Discussion sections were not detected from the experimental blanks. It is possible that carryover of abundant peptides across the injection blank in the LC column resulted the detection of some peptides in experimental blanks (Hendy et al., 2018).

(b) Comparison with modern dog bone proteome

Comparison showed that 23 out of 83 protein groups (27.7%) detected in a modern dog bone (Cleland, 2018) were shared with the protein groups detected in this study. PANTHER analysis indicated that the protein groups that are detected only in modern adult dogs (Cleland, 2018) or archaeological neonatal dogs (this study) bones showed similar compositions in terms of biological process, molecular function, and cellular component (Supplementary Figure 3).

Proteomic differences between the modern adult dog femur (Cleland, 2018) and the ancient neonatal dog rib and vertebra (this study) (Supplementary Table 2 and 6) likely originate from

differences in bone elements, biological ages of the individuals, and differences in protein extraction methods. Bone samples from different parts of the skeleton (Procopio et al., 2017) and different biological ages (Procopio et al., 2017; Sawafuji et al., 2017) indicate different proteome composition, and these differences are mostly driven by a different status in the physiological process of bone ossification and elongation (Procopio et al., 2017). Although most bones of adult dogs have ossified, the rib and vertebra bones of 2017HA1016 did not complete its ossification. Protein extraction methods used differed between the previous study (Cleland, 2018) and this study: the EDTA fraction was not completely analyzed and collagen fibrils were not denatured with heat in the previous study (Cleland, 2018). These methodological differences could cause the proteomic difference between them (e.g., Cersoy et al., 2019; Cleland et al., 2012; Schroeter et al., 2016; Wadsworth and Buckley, 2018).

(c) Possibility of contamination by bacteria

Only two protein groups were identified from the bacterial database, and the original peptide-spectrum matches of LGB1 and WAP were obtained from the dog bone fractions when the RAW files were searched against Swiss-Prot bacterial protein and the UniProt dog proteome databases (Supplementary Table 7). This result suggests that the detected peptide-spectrum matches of LGB1 and WAP were not the result of contamination of bacterial proteins.

References

Cersoy S, Zirah S, Marie A, Zazzo A. 2019. Toward a versatile protocol for radiocarbon and proteomics analysis of ancient collagen. J Archaeol Sci 101:1–10.

Cleland TP, Voegele K, Schweitzer MH. 2012. Empirical evaluation of bone extraction protocols. PLoS ONE 7:e31443.

Cleland TP. 2018. Solid digestion of demineralized bone as a method to access potentially insoluble proteins and post-translational modifications. J Proteome Res 17:536–542.

Cox J, Mann M. 2008. MaxQuant enables high peptide identification rates, individualized p.p.b.-range mass accuracies and proteome-wide protein quantification. Nat Biotechnol 26:1367–1372.

Hendy J, Welker F, Demarchi B, Speller C, Warinner C, Collins MJ. 2018. A guide to ancient protein studies. Nat Ecol Evol 2:791–799.

Mackie M, Rüther P, Samodova D, Di Gianvincenzo F, Granzotto C, Lyon D, Peggie DA, Howard H, Harrison L, Jensen LJ, Olsen JV, Cappellini E. 2018. Palaeoproteomic profiling of conservation layers on a 14th century Italian wall painting. Angew Chemie 57:7369–7374.

Mi H, Muruganujan A, Casagrande JT, Thomas PD. 2013. Large-scale gene function analysis

with the PANTHER classification system. Nat Protoc 8:1551–1566.

Procopio N, Chamberlain AT, Buckley M. 2017. Intra- and interskeletal proteome variations in fresh and buried bones. J Proteome Res 16:2016–2029.

Rappsilber J, Mann M, Ishihama Y. 2007. Protocol for micro-purification, enrichment, pre-fractionation and storage of peptides for proteomics using StageTips. Nat Protoc 2:1896–1906.

Sawafuji R, Cappellini E, Fotakis AK, Rakownikow R, Olsen J V, Hirata K, Ueda S. 2017. Proteomic profiling of archaeological human bone. R Soc Open Sci 4:161004.

Schroeter ER, DeHart CJ, Schweitzer MH, Thomas PM, Kelleher NL. 2016. Bone protein "extractomics": comparing the efficiency of bone protein extractions of *Gallus gallus* in tandem mass spectrometry, with an eye towards paleoproteomics. PeerJ 4:e2603.

Van Doorn NL, Wilson J, Hollund H, Soressi M, Collins MJ. 2012. Site-specific deamidation of glutamine: a new marker of bone collagen deterioration. Rapid Commun Mass Spectrom 26:2319–2327.

Wadsworth C, Buckley M. 2018. Characterization of proteomes extracted through collagen-based stable isotope and radiocarbon Dating Methods. J Proteome Res 17:429–439.

Supplementary Figures

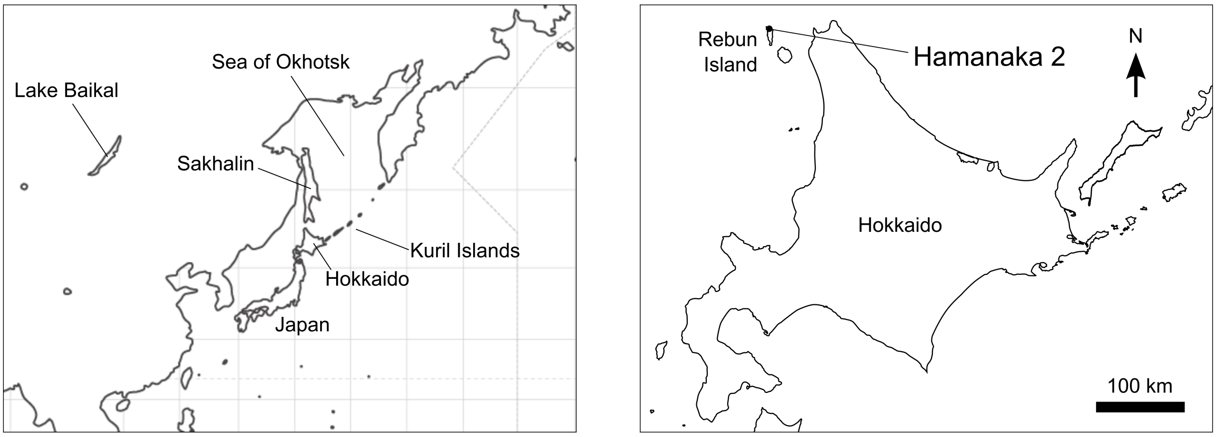

Supplementary Figure 1. Map of the location of Hamanaka 2 site.

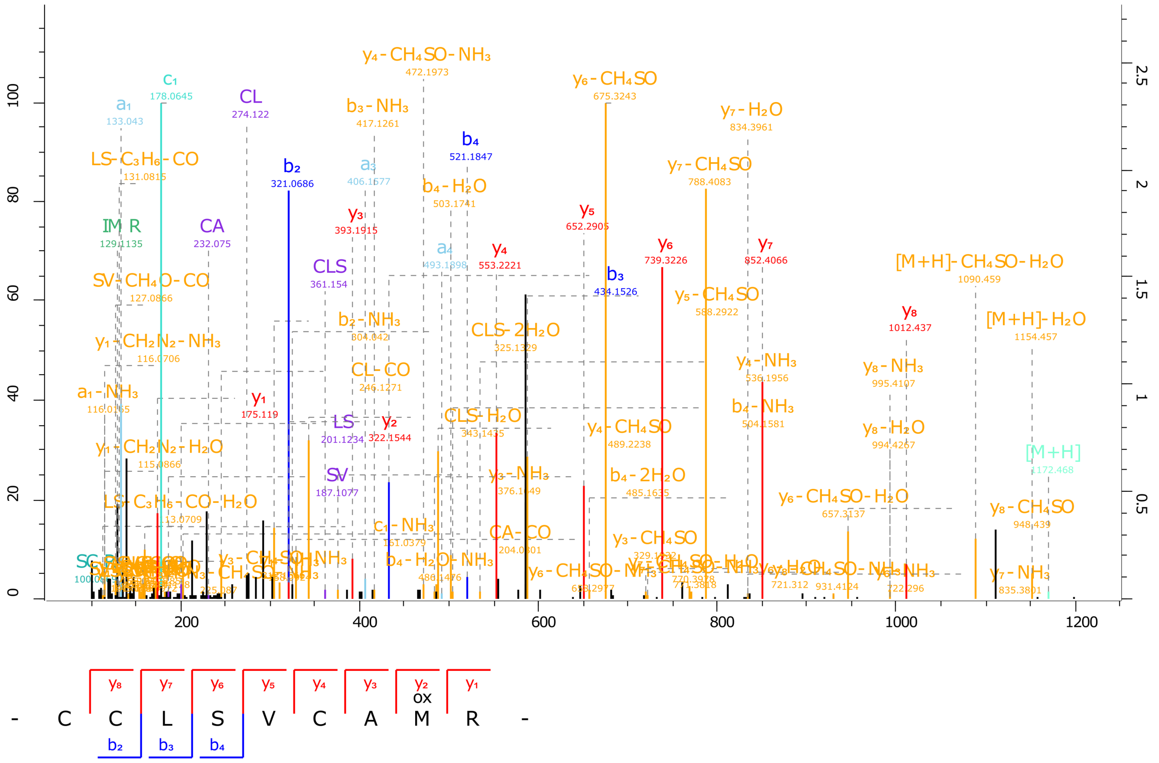

Supplementary Figure 2. An annotated MS2 spectrum for the WAP peptide (CCLSVCAMR) that were detected in 2017HA1016.

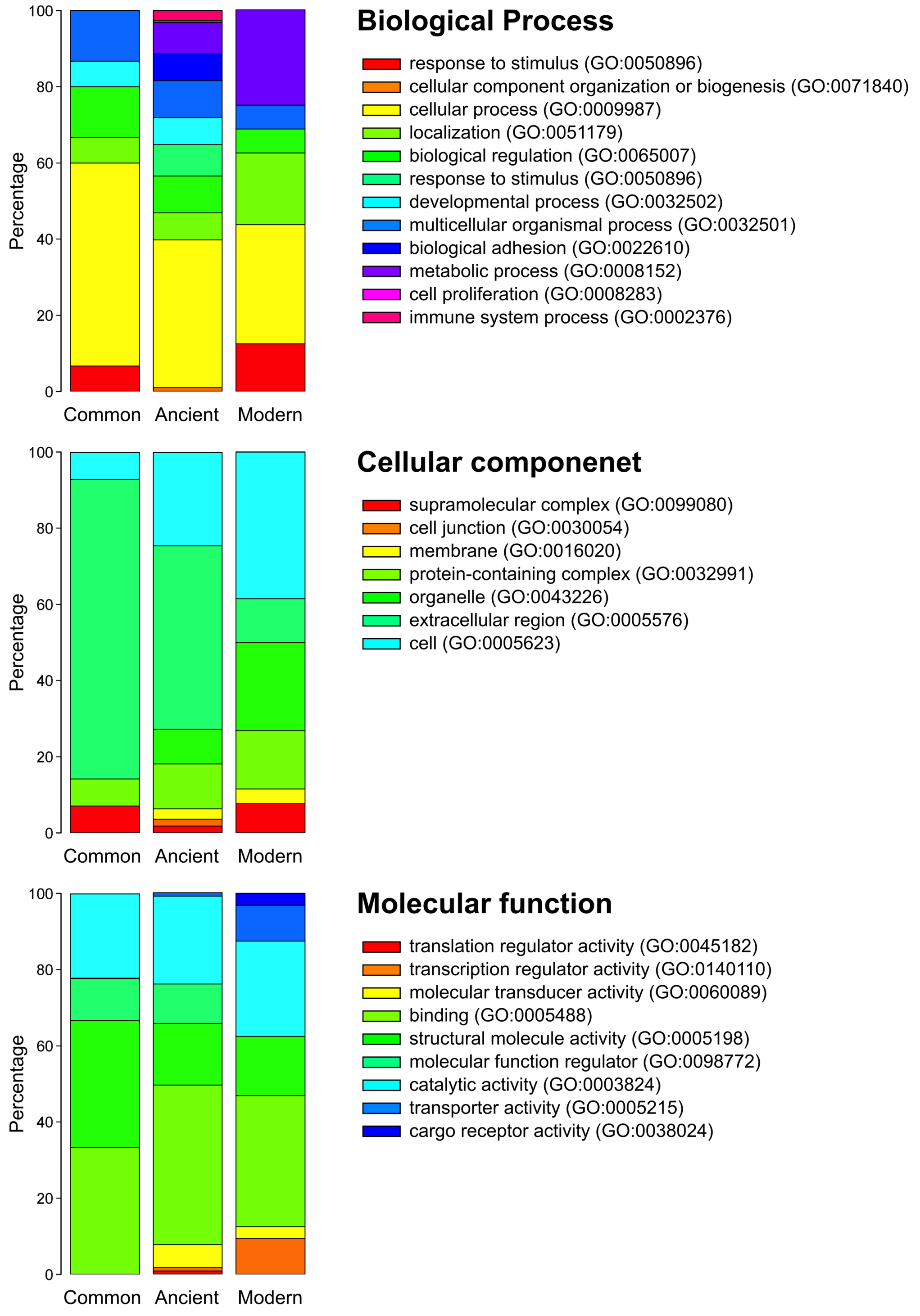

Supplementary Figure 3. Results of PANTHER biological process, molecular function, and cellular component analysis of protein groups that are unique to ancient neonatal dog ribs and vertebra (this study) and modern adult dog femur (Cleland, 2018).

Supplementary Tables

Supplementary Table 1. List of recovered bones of 2017HA1016.

Skeletal part	Element	Side	Number
Cranium	Maxilla	L	1
		R	1
	Frontal	L	1
		R	1
	Parietal	R	2
		L	1
		?	2
	Petrous part of temporal	R	1
	Fragment of temporal	?	1
	Exoccipital	L	1
	Occipital condyle	R	1
	Fragment	–	6
Limb	Scapula	R	1
	Humerus	L	1
	Radius	R	1
	Femur	R	1
	Tibia	R	1
Other	Rib	–	Many
	Vertebra	–	Many

Supplementary Table 2. List of detected protein groups from 2017HA1016. Protein groups that are shared with modern adult dog humerus (Cleland, 2018) have a tick mark in the 'Common' column. Protein groups identified in blanks but not in dog bones with ≥2 peptides have a tick mark in the 'Only identified in blank' column. Sample names correspond with those shown in Table 1 along with the experimental blanks (BE, BP, B1, and B2).

[supplementary_table_2.csv]

Supplementary Table 3. Result of PANTHER cellular component analysis of proteins detected in 2017HA1016.

Cellular Component	Gene ontology ID	Number	Percent
Extracellular region	GO:0005576	64	51.6
Cell	GO:0005623	28	22.6
Protein-containing complex	GO:0032991	14	11.3
Organelle	GO:0043226	10	8.1

Supramolecular complex	GO:0099080	3	2.4
Membrane	GO:0016020	3	2.4
Cell junction	GO:0030054	2	1.6

Supplementary Table 4. Result of PANTHER molecular function analysis of proteins detected in 2017HA1016.

Molecular Function	Gene ontology ID	num	percent
Binding	GO:0005488	52	41.3
Catalytic activity	GO:0003824	29	23.0
Structural molecule activity	GO:0005198	22	17.5
Molecular function regulator	GO:0098772	13	10.3
Molecular transducer activity	GO:0060089	7	5.6
Translation regulator activity	GO:0045182	1	0.8
Transcription regulator activity	GO:0140110	1	0.8
Transporter activity	GO:0005215	1	0.8

Supplementary Table 5. Calculated deamidation rates for the samples of 2017HA1016.

Experimental ID	Amino acid	Mean	SD	95% CI low	95% CI up
1016V-E	N	34.4	2.3	29.5	38.9
	Q	7.3	1.0	5.4	9.1
1016V-P	N	28.1	1.9	24.1	31.6
	Q	9.1	1.0	7.0	11.0
1016R1d-E	N	40.0	2.8	34.2	45.4
	Q	11.8	1.3	9.2	14.4
1016R1d-P	N	35.6	2.2	31.1	39.7
	Q	9.6	1.1	7.4	11.6
1016R1p-E	N	43.5	1.7	40.1	46.9
	Q	12.7	0.9	10.8	14.5
1016R1p-P	N	32.2	3.2	25.8	38.3
	Q	11.7	1.8	8.4	15.1
1016R2	N	38.6	2.7	33.0	43.6
	Q	12.9	1.6	9.9	16.0
1016R3	N	37.7	3.5	30.7	44.0
	Q	13.1	2.0	9.4	17.2
Total mean	N	36.3	4.8	–	–

Q	11.0	2.1	–	–

Supplementary Table 6. List of detected protein groups from the dataset of Cleland (2018). Protein groups that are shared with ancient neonate dog rib or vertebra (this study) have a tick mark in the Common column. E, EDTA fraction; SD, solid digested pellet fraction with reduction and alkylation; NRA, solid digested pellet fraction without reduction and alkylation.

[supplementary_table_6.csv]

Supplementary Table 7. List of detected protein groups from the EDTA fractions of two subsamples of 2017HA1016's rib bone 1 and the soil sample by searching against a Swiss-Prot bacterial proteins and a UniProt dog proteome. Sample names correspond with those shown in Table 1.

[supplementary_table_7.csv]
